# Rationality, the Bayesian standpoint, and the Monty-Hall problem

**DOI:** 10.3389/fpsyg.2015.01168

**Published:** 2015-08-11

**Authors:** Jean Baratgin

**Affiliations:** ^1^Laboratory CHArt (PARIS), Université Paris 8Paris, France; ^2^Institut Jean NicodParis, France

**Keywords:** Bayesian standpoint, Monty-Hall problem with two players, probability revision, collider principle, single case probability

## Abstract

The Monty-Hall Problem (*MHP*) has been used to argue against a subjectivist view of Bayesianism in two ways. First, psychologists have used it to illustrate that people do not revise their degrees of belief in line with experimenters' application of Bayes' rule. Second, philosophers view *MHP* and its two-player extension (*MHP*_2_) as evidence that probabilities cannot be applied to single cases. Both arguments neglect the Bayesian standpoint, which requires that *MHP*_2_ (studied here) be described in different terms than usually applied and that the initial set of possibilities be stable (i.e., a focusing situation). This article corrects these errors and reasserts the Bayesian standpoint; namely, that the subjective probability of an event is always conditional on a belief reviser's specific current state of knowledge.

## 1. Introduction

In *the Monty Hall Problem* (*MHP*), you know that the car you want is behind one of three closed doors and a goat behind the other two doors. You choose a door and Monty (the host who knows where the car is) opens another door with a goat behind (as you know he can neither open your door nor a door with the car behind). After the host's action, would you rather stick to your original choice or switch to the remaining door?

*MHP* is a much-studied experimental paradigm investigating the inability of (naive and expert) people to revise their degrees of belief in a Bayesian manner (for a recent review see Tubau et al., 2015). Specific reformulations of format (natural frequencies, nested sets, visual representation, etc.) improving Bayesian performance have triggered some psychological debates on the underlying cognitive processes at play (for a recent analysis see Brase and Hill, 2015). Baratgin (2009) argues that these different formats facilitating Bayesian performance actually enhance the correct representation of the situation of revision in a stable universe, called the situation of *focusing* (Dubois and Prade, 1992, 1997) for which only Bayes' rule applies. The standard formulation of *MHP* prompts participants to form different representations of the situation of revision. However, when participants perceive the situation of focusing (for instance in a disambiguated version of *MHP* as in Baratgin and Politzer, 2010), they produce the Bayesian answer. Hence, participants cannot be considered as incoherent but only prone to an error induced by experimenters' presentation (Baratgin, 2009; Baratgin and Politzer, 2010).

*MHP* is also used as an argument against the notion of single-case probabilities. Moser and Mulder (1994) argued that there existed two opposite rational solutions: “sticking” for a *MHP* proposed as a one-shot problem and “switching” for a *MHP* cast in a frequentist context (i.e., when imagining a sufficiently large number of games). Horgan (1995) opposed this view making explicit the correct solution for the one shot *MHP* and showing that switching is the only correct solution to both formulations. Baumann (2005, 2008) produced a new argument based on a generalization of *MHP*: *the Monty Hall Problem with two players* (*MHP*_2_, see Table 1). In his view, although the two players share the same initial state of knowledge, they eventually form two different probability distributions. This point of view is opposed by Levy (2007) and by Sprenger (2010) who rightly argue that the two players do not necessarily share the same state of knowledge *throughout* the game in particular when their original choices differ. However, these authors do not explain the rationale of Baumann's mistake and do not explicitly define the causal structure of *MHP*_2_^1^.

**Table 1:** **The six sequential stages of *MHP*_2_**.

**Stages**	**Descriptions**
Stage 1	The TV host shows to two players (players *A* and *B*) three identical doors (let them be *D*_1_, *D*_2_, and *D*_3_) all equally likely, one hiding a car and the other two hiding goats. It is assumed that the host has no preference for a specific door when he initially places the car behind it and that both players prefer to win the car than a goat.^a^ It is also assumed that the two players have a common initial state of knowledge and that no player has any preference for a particular door. The players fully grasp the six stages of *MHP*_2_ and accept the implicit and explicit rules implied by its statement.
Stage 2	Each player picks a door and neither player is informed of the other player's choice. Let's assume for the sake of convenience that you are player *A* and you initially select door *D*_1_.
Stage 3	The host, who knows where the car is, tells you: “In the case where player *B* has chosen the same door as you (here *D*_1_), I will show you one door (out of the two other doors) behind which there is a goat.” It is assumed that both players know that the host has no preference between the two remaining doors (*D*_2_ and *D*_3_) to show a goat should the car be behind *D*_1_. Then the host continues: “In the case where player *B* has picked another door, I will always open the third door -chosen by neither player- even if the car is behind it.” In this latter case when the host reveals a car, both players (you and player *B*) win and have no decision to make; the game stops.
Stage 4	The host says “I will open a door to reveal a goat” and then asks both players still ignorant of the other player's original choice: “To win the car should you stick to your original choice or switch to another door (as far as you are concerned door *D*_2_ or door *D*_3_).”
Stage 5	The host opens a door (for example *D*_3_), reveals a goat and then asks both players again: “To win the car, should you stick to your original choice or switch to the other closed door (door *D*_2_ in your case)?”
Stage 6	Each player reveals her or his original choice and must then decide knowing the other player's choice whether to stick to her/his door (*D*_1_ in your case) or to switch door (*D*_2_ in your case)^b^.

a In the case where both players succeed in their door choice with the car, they each get a car. Hence, as noted by Sprenger (2010), there is no real competition between both players.

b This version of MHP_2_ is derived from Baumann's version (Baumann, 2005). The transitional Stage 4 is not presented by Baumann but it interestingly draws a comparison with MHP where this information is not informative. We also added the Stage 6 to find again MHP in the situation where the two players have originally chosen the same door.

This paper will address these questions. First, the solution to *MHP*_2_ proposed as a one shot and its causal structure will be detailed. Then, explanations for the failure of researchers investigating *MHP*_2_ will be advanced and related to the “bias” that conducts psychologists to wrongly conclude that participants' responses to *MHP* are of a non-Bayesian nature, that is, the *neglect of the Bayesian standpoint* (de Finetti, 1974).

## 2. Solving the monty hall problem with two players

Let's consider the following variables that define the properties of the possible doors (*D*_1_,*D*_2_,*D*_3_) in *MHP*_2_: The three variables *C* (*The host's original choice of the door in which to place the car*), *Y* (*Your original choice of door*) and *B* (*Player B*'*s original choice of door*). *C*, *Y*, and *B* can take any of the three values *D*_*i*_ (with *i* ∈ {1, 2, 3}), respectively noted from now on *c*_*i*_, *y*_*i*_, and *b*_*i*_. The variable *H* (*the host's choice when opening a door*) is composed of the two complementary sub variables ‘*G*’ (*the host's revealing a goat*) and ‘*C*’ (*the host's revealing a car*). The sub variables ‘*G*’ and ‘*C*’ can take the three values *D*_*i*_ (with *i* ∈ {1, 2, 3}), respectively noted from now on ‘*g*_*i*_’ and ‘*c*_*i*_’^2^.

**Figure 1:**
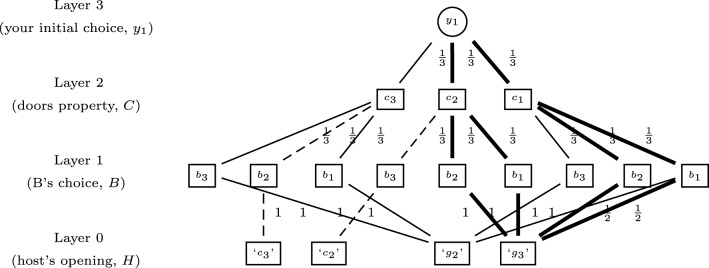
**The general tri-probabilistic structure of *MHP*_2_ before learning message ‘*g*_3_’ assuming your initial choice is *D*_1_ (*Y* = *y*_1_)**. The continuous lines correspond to the subset left after compiling information at Stage 4 and the bold lines to the subset left after compiling the information at Stage 5. Conversely the dashed lines represent the initial structure dropped out at Stage 4.

Following Walliser and Zwirn (2011), your beliefs before learning message ‘*g*_3_’ assuming your initial choice is *D*1 (Stage 2) can be represented as a hierarchical dynamic probabilistic structure (see Figure 1). The layer 0 depicts the four possible strategies of the host, i.e., showing a goat behind *D*_2_ or *D*_3_ (‘*g*_2_’ or ‘*g*_3_’) or showing a car when the two players have originally chosen two different doors with goats behind (‘*c*_2_’ or ‘*c*_3_’). Layer 1 corresponds to the three possible original choices of player B (*b*_1_, *b*_2_ or *b*_3_). Layer 2 represents the original car placement choice of the host (*c*_1_, *c*_2_, or *c*_3_). Layer 3 is your original choice (*y*_1_). The probability distributions of the variables at the different layers are defined by the statement of *MHP*_2_ with implicit and explicit hypotheses about the host's action and the players' preferences.

At Stage 4 you learn that the host will open a door with a goat behind. You know that (i) this door is either door *D*_2_ or *D*_3_ and (ii) the car is either behind your door *D*_1_ or player *B*'s originally chosen door. Hence you focus on the subset where ‘*g*_2_’ or ‘*g*_3_’ is true (the continuous lines in Figure 1). You are better off sticking to your initial choice *D*_1_.

(1) $$P(c_{1}|y_{1}\text{‘}G\text{’})=3/7>2/7=P(c_{2}|y_{1}\text{‘}G\text{’})=P(c_{3}|y_{1}\text{‘}G\text{’})$$

Second at Stage 5 the host opens door *D*_3_ and reveals a goat behind. You focus on the subset where ‘*g*_3_’ is true (the bold lines in Figure 1). This information combined with your original choice of door provides information about the door behind which Monty placed the car. You are better off switching to door *D*_2_.

(2) $$P(c_{1}|y_{1}\text{‘}g_{3}\text{’})=3/7<4/7=P(c_{2}|y_{1}\text{‘}g_{3}\text{’})$$

Finally at Stage 6 you learn what was player *B*'s original choice. On the one hand, it can coincide with yours (*b*_1_). Both players are then exactly in the same situation with the same common knowledge. *MHP*_2_ amounts to *MHP*. Hence, you know that *C* is twice as likely to have the value *c*_2_ as to have the value *c*_1_. The best strategy is to switch from your original choice to the other closed door *D*_2_.

(3) $$P(c_{1}|y_{1}b_{1}\text{‘}g_{3}\text{’})=1/3<2/3=P(c_{2}|y_{1}b_{1}\text{‘}g_{3}\text{’})$$

On the other hand you may learn that player *B*'s original choice is different from yours (*b*_2_). In this case there is no best strategy and you are indifferent to sticking or switching.

(4) $$P(c_{1}|y_{1}b_{2}\text{‘}g_{3}\text{’})=1/2=P(c_{2}|y_{1}b_{2}\text{‘}g_{3}\text{’})$$

## 3. The collider principle

Glymour (2001) was the first to identify the causal structure in *MHP* as a situation where two independent variables that mutually influence another variable are dependent conditional on the value of the variable they both affect. In *MHP*_2_, the three independent variables *Y*, *B*, and *C* symmetrically influencing (colliding with) another variable *H* (common effect) actually appear dependent conditionally on the values of the variable *H*. Hence observing the value of *H* provides some information on the possible values of *Y*, *B* or *C*. In the same way, knowing the values of any couple of variables (*C*, *H*), (*B*, *H*), and (*Y*, *H*) provides some information about the values of couples (*Y*, *B*), (*Y*, *C*), and (*B*, *C*), respectively. Finally observing the values of triples (*Y*, *C*, *H*), (*B*, *C*, *H*), (*Y*, *B*, *H*), respectively determines the values of variables *B*, *Y*, and *C*. Solving *MHP*_2_ as a one shot game relies on the latter triple (*Y*, *B*, *H*). It is easy when two variables are fixed to derive some qualitative predictions (Wellman and Henrion, 1993). For instance, *MHP*_2_'s solution supports a phenomenon of reversal decision resulting from this collider principle. On learning *H* = ‘*g*3’ given your original choice (*Y* = *y*_1_) the likelihoods that *B* and *C* equal *b*_2_ and *c*_2_, respectively, are higher than the likelihoods that *B* and *C* equal *b*_1_ and *c*_1_, respectively. However, if in addition you learn that *B* equals *b*_1_ then the outcome *c*_2_ seems the more likely. However, if you learn that *B* equals *b*_2_ then the probabilities for the car being behind either *D*_1_ or *D*_2_ are even.

Recent studies have provided some evidence that “naive” adults and also children make correct qualitative predictions in collider principle situations when pairs of causal conditionals are explicitly presented (Ali et al., 2010, 2011). Precisely in *MHP*, participants perform better when the relation between the player's original choice and the host's strategy is explicit in conditional form (Macchi and Girotto, 1994, cited in Johnson-Laird et al., 1999). In the same way, when participants can construct a representation analogous to Figure 1 for *MHP* using a graph or by means of physical handling, participants' performance improves significantly (Yamagishi, 2003; Baratgin and Politzer, 2010). Thus, it seems that when participants can infer the causal structure of *MHP* by physical or explanatory cues, they are able to solve *MHP* (Burns and Wieth, 2004; Chater and Oaksford, 2006).

## 4. The neglect of the bayesian standpoint

De Finetti's subjective Bayesian standpoint proposes that individuals form two levels of knowledge (de Finetti, 1980; Baratgin and Politzer, in press):

- An elementary level of knowledge of an event *E* that is always conditioned on an individual's specific state of knowledge {*H*_0_} at this time. Furthermore, any event is actually a tri-event (the third value representing ignorance between true event and false event).
- A meta-level of knowledge concerning the degrees of belief of an individual. Here ignorance is specified, and refined, into degrees of belief. From an inferential point of view, your subjective probability of this event *E* at time *t*_0_ is always *conditional on your current state of knowledge* {*H*_0_} [and should be written *P*(*E*|*H*_0_)]. It is *coherent* if (i) it follows the axiom of additive probabilities^3^ and (ii) when acquiring a new knowledge *H*, your probability also depends on this new knowledge {*H*_0_*H*} [and should be written *P*(*E*|*H*_0_*H*)].

A person dismissing the Bayesian standpoint considers the probability of a single event as questionable as compared to a “frequentist” conception of probability. She takes the frequentist conception to be the “correct” comparative representation, and confines Bayesianism to just a set of *Bayesian techniques* (de Finetti, 1974). In the psychological literature this “bias” leads to two significant mistakes: (i) to the neglect of pragmatic constraints on the methodology (to understand *H*_0_ and *H*); (ii) to the conclusion that people's behavior is “non-Bayesian,” even when the behavior does not violate Bayesian coherence (Baratgin, 2002; Mandel, 2014a). In the analysis of *MHP*_2_, this bias is characterized by inadequate terminology and interpretation of the revision situation.

### 4.1. The use of an “Ambiguous Terminology”

For a subjective Bayesian, an event *E* always refers to a certain outcome in a single well-defined case (a unit in which the definition is unambiguous and complete) and cannot be used in a generic sense (such as a collection of “identical events”). There is no repetition of the same event but a succession of many distinct events, which can be different illustrations of the same phenomenon. In Moser and Mulder (1994), Baumann (2005), Levy (2007), and Baumann (2008), *MHP*_2_ is presented in an *ambiguously termed* way (de Finetti, 1977/1981, p. 357). The variables are considered as trials of the same phenomenon without completely specifying them and their possible values. Every specific door corresponds to a generic door *D* that is characterized by two properties: having a car (*C*) or a goat (*G*) behind it. Every player's original door choice is analyzed by its correspondence with *C* and *G*. The host's door opening ‘*H*’ is characterized by the two sub-classes ‘*G*’ and ‘*C*’. The players' final decisions to win the car are commingled and considered to pertain to the same classes of events “to stick,” “to switch” or “nothing.”

Following this *frequentist “jargon”* (de Finetti, 1979a,b), *MHP*_2_ is analyzed as an observation of a repetitive problem where the different variables are interchangeable in function of the host's car placement. Instead of considering each player with specific states of knowledge relative to each stage of *MHP*_2_ both players are assumed to have a *common knowledge* at each stage of the game. Their probabilities that there is a car behind one of the two remaining doors (after the door with a goat behind was opened) is 3∕7 for the door originally chosen and 4∕7 for the other door. Thus, imagining they made a different original choice, each door can be associated with two different probabilities (3∕7 and 4∕7) illustrating Bauman's paradox. Now, if we consider the specific knowledge of each player, the paradox disappears. In Stages 4 and 5, player *B*'s probabilities on *c*_1_ and *c*_2_ are identical to your probabilities (relations 1–3) when his/her specific initial state knowledge is identical to yours (his/her original choice is *b*_1_). Conversely when his/her original choice is *b*_2_, his/her state of knowledge is different from yours and his/her probabilities correspond to different probabilities (relations 5 and 6):

(5) $$P(c_{1}|b_{2}\text{‘}G\text{’})=P(c_{3}|b_{2}\text{‘}G\text{’})=2/7<3/7=P(c_{2}|b_{2}\text{‘}G\text{’})$$

(6) $$P(c_{1}|b_{2}\text{‘}g_{3}\text{’})=4/7>3/7=P(c_{2}|b_{2}\text{‘}g_{3}\text{’})$$

However, player *B*'s decisions are identical: sticking at Stage 4 and switching at Stage 5. At Stage 6, both players have an identical state of knowledge and probabilities (relation 4).

### 4.2. Neglect of the situation of focusing

*MHP*_2_ illustrates that the situation of revision implied by the Bayesian standpoint is a process of *focusing* on a subset of the initial state of knowledge {*H*_0_} (de Finetti, 1957; Dubois and Prade, 1992, 1997). It is assumed that one object is selected from the universe and that a message releases information about it. A reference class different from the initial one is consequently considered by *focusing* attention on a given subset of the original set that complies with the information about the selected object. This is not a temporal revision process because the information ‘*g*_3_’ just focuses on the selection of a particular posterior probability that was virtually available (among others) (see the bold lines of Figure 1). Yet participants in *MHP* seem to adopt (for pragmatic reasons) another representation of the revision situation, known as *updating* (Katsuno and Mendelzon, 1992; Walliser and Zwirn, 2002) in which, they infer from the message ‘*g*_3_’ the information as “door *D*_3_ have been removed,” and conceive a new probability distribution consistent with this *new problem* (Baratgin and Politzer, 2007, 2010; Baratgin, 2009). In this representation there is obviously no collider effect because, in this *new problem with two doors*, the variables *Y* and *H* always remain independent after the information is provided by the host. Participants form a new distribution of probability *P*′ for this new game^4^. Two typical analyses are consistent with this interpretation:

The *stick or switch response*: if you originally chose door *D*_1_ and the host opens door *D*_3_ with a goat behind, the worlds *c*_1_ and *c*_2_ are evenly close (in fact proportionally to their prior probabilities) to the invalidated world *c*_3_. The weight of *c*_3_ is redistributed proportionally on *c*_1_ and *c*_2_. This is *MHP*'s solution in the updating context proposed by Dubois and Prade (1992).

(7) $$\begin{array}{l} P\text{‘}(c_{1}|y_{1})=P(c_{1}|y_{1})+1/2P(c_{3}|y_{1})=1/2\\ \;=P(c_{2}|y_{1})+1/2P(c_{3}|y_{1})=P\text{’}(c_{2}|y_{1}) \end{array}$$

It corresponds to the “equiprobability” solution given by nearly all participants to *MHP* but also by some experts in their analysis of *MHP* in a single isolated situation (Moser and Mulder, 1994) and of *MHP*_2_ (Levy, 2007).

The *switch response*: The worlds *c*_3_ and *c*_2_ (the two doors not originally chosen by the player) are considered closer. The probability of the invalidated world *c*_3_ is transferred to *c*_2_ alone. This is *MHP*'s solution in the updating context proposed by Cross (2000).

(8) $$\begin{array}{l} P\text{‘}(c_{1}|y_{1})=P(c_{1}|y_{1})=1/3\;\text{and}\;\\ P\text{’}(c_{2}|y_{1})=P(c_{2}|y_{1})+P(c_{3}|y_{1})=2/3 \end{array}$$

This response is given by only few participants to *MHP* (see for review Baratgin, 2009). It corresponds to Moser and Mulder's explanation for *MHP*'s solution in a suitable long run of relevantly similar situations. To explain the “causal structure” of *MHP*, Levy (2007) proposed also a process in line with this updating interpretation. However, it is difficult here to support the “switch” response to *MHP*_2_ with the symmetric role of the two players (Levy, 2007). Thus, the “stick or switch response” should be privileged to solve *MHP*_2_ in an updating representation.

## 5. Conclusion

This paper describes the supposedly paradoxical solutions attributed to *MHP*_2_ from the perspective of a thorough Bayesian standpoint perspective. It outlines the methodological care that one should take to comprehend the problem in relation to the single case terminology and the focusing context of revision. Not taking into account these features prevents one from fully grasping the probabilistic temporal dynamics of the problem and consequently the corresponding causal collider structure.

Psychologists who study subjective Bayesian reasoning should carefully formulate the statement without ambiguity and respect the Bayesian standpoint. This is also true especially for complex problems (such as the Sleeping Beauty problem Baratgin and Walliser, 2010; Mandel, 2014b) in which different solutions can be envisaged depending on the interpretations made by participants.

### Conflict of interest statement

The author declares that the research was conducted in the absence of any commercial or financial relationships that could be construed as a potential conflict of interest.

## Footnotes

^1^The term “causal” is missing in Baumann (2005). We find Horgan's terminology of “causal structure” in Levy (2007) with the vague definition of: “the set of conditions that ultimately explains why sticking and switching have the probabilities that they do” (Levy, 2007, p. 146). Finally, Sprenger (2010, p. 337) admits that “the place of causality in the ‘causal structure’ of a Monty Hall game remains obscure.”

^2^We use here quotes for all sub-variables related to the host's actions during the game.

^3^See for example on this special research topic (Cruz et al., 2015; Evans et al., 2015; Mandel, 2015) and also (Politzer and Baratgin, in press).

^4^*P*′ along the following process: (i) The worlds ‘*c*_3_’ and ‘*g*_2_’ are canceled and a simpler probabilistic structure composed of the two worlds (*c*_1_, *c*_2_) is obtained, (ii) The new distribution *P*′ stems from a redistribution of the weights (the probabilities) of the removed worlds on the two remaining worlds.
